# Development of a national consensus guideline for termination of resuscitation in the out-of-hospital environment

**DOI:** 10.29045/14784726.2026.6.11.1.56

**Published:** 2026-06-01

**Authors:** Michael A. Smyth, Terry P. Brown, Rachael Fothergill, Frances Griffiths, Ranjit Lall, John Long, Stavros Petrou, Aloysius Niroshan Siriwardena, Anne-Marie Slowther, Lauren Bettely, Karin Eli, Galina Gardiner, Caroline Huxley, Kamran Khan, Felix Michelet, Gavin D Perkins

**Affiliations:** University of Warwick; University Hospitals Coventry & Warwickshire NHS Trust; University of Warwick; University of Warwick; London Ambulance Service NHS Trust; University of Warwick; University of Warwick; University of Warwick; University of Oxford; University of Lincoln; University of Warwick; University of Warwick; University of Warwick; University of Warwick; University of Warwick; University of Warwick; University of Warwick; University of Warwick; University Hospitals Birmingham NHS Foundation Trust

**Keywords:** ambulance, emergency medical services, guideline, paramedic, pre-hospital, termination of resuscitation, verification of death

## Abstract

**Introduction:**

Ambulance services in England attempted resuscitation in over 34,400 cases in 2022. Of these, 58% had the resuscitation attempt terminated at the scene and only 7.8% survived to hospital discharge. The decision to stop resuscitation is informed by a national guideline that is over 20 years old. This study describes the development of a revised termination of resuscitation (TOR) guideline.

**Methods:**

This was a mixed-methods study comprising a diagnostic test accuracy meta-analysis of TOR rules, modelling of multiple TOR rules using data from the Out-of-Hospital Cardiac Arrest Outcomes registry, a survey of ambulance services including a review of national policy documents, qualitative interviews with ambulance and emergency department (ED) clinicians, plus interviews with relatives of patients who did not survive a pre-hospital resuscitation attempt. These work packages informed a national consensus meeting with a wide range of stakeholders, employing nominal group techniques, to draft a revised TOR guideline.

**Results:**

The systematic review identified very low-certainty evidence from 43 studies, indicating that TOR rules are unlikely to be suitable for implementation in the UK. When we modelled the performance of TOR rules, the three best performing were the Marsden, KOCARC 1 and GOTO1 TOR rules. We identified considerable variation in practice across UK ambulance services; however, there was consistency across services with respect to perceived risks. Paramedics experienced tension when they felt that guidelines restricted them from acting in the patient’s best interests. ED staff felt that paramedics should be empowered to stop resuscitation in some cases. Relatives felt that paramedics did a good job and that they had information that was useful for paramedics. Multiple stakeholders participated in a consensus conference to develop a revised TOR guideline.

**Conclusion:**

We iteratively derived updated TOR and verification of death guidelines.

## Introduction

In 2022 National Health Service (NHS) Ambulance Services in England responded to over 98,000 calls for out-of-hospital cardiac arrest (OHCA). Of these patients, 58.3% were declared dead on scene, 15.9% were transported to hospital with ongoing cardiopulmonary resuscitation (CPR) and 25.8% were admitted to hospital with return of spontaneous circulation (ROSC). Ultimately, only 7.8% of patients survived to 30 days (Out-of-Hospital Cardiac Arrest Outcomes (OOHCA) Registry, 2023).

In the UK the decision to stop resuscitation or to transport with ongoing resuscitation is informed by a national verification of death guideline (Joint Royal Colleges Ambulance Liaison Committee (JRCALC), 2021). The guideline was written by the Guidelines Sub-committee of the Joint Royal Colleges Ambulance Liaison Committee (JRCALC) and approved by the National Ambulance Service Medical Directors group (NASMeD) on behalf of the Association of Ambulance Service Chief Executives (AACE). The guideline has been in existence for over 20 years and was originally called the Recognition of Life Extinct (ROLE) guideline. It was based upon expert consensus published by the European Resuscitation Council (ERC) (Mentzelopoulos et al., 2021). The verification of death guideline has been subject to several modifications over the past 20 years. Notable changes include the duration of resuscitation required and further detail addressing termination in pulseless electrical activity (PEA). However, the core guideline principles have remained largely unchanged.

The verification of death guideline used by ambulance services has never been subject to formal evaluation. Recent evidence concerning the utility of end-tidal carbon dioxide (EtCO_2_) trend (Engel et al., 2019), duration of resuscitation (Goto et al., 2016; Nagao et al., 2013) and the impact of transport on patient outcomes (Grunau et al., 2017a) challenges some of the assumptions within the verification of death guideline. The Exploring and Improving Resuscitation Decisions in Out-of-Hospital Cardiac Arrest (PROTECTeD) study (NIHR 17/99/34) sought to develop an updated national termination of resuscitation (TOR) guideline (Supplementary 1), that could be adopted across the ambulance service, in order to improve out-of-hospital TOR decisions.

## Methods

This was a mixed-methods study to develop an evidence-based TOR guideline, in collaboration with multiple stakeholders.

Key objectives of the study were to:

- Evaluate how ambulance services implemented the existing verification of death guideline.
- Better understand where there was consistent and variable practice and to identify areas of perceived increased risk.
- Review existing guidelines and policy documents pertaining to TOR, including a content analysis to identify content not present in the verification of death guideline.
- Complete a diagnostic test accuracy meta-analysis of published TOR rules.
- Model the performance of identified TOR rules using data from the national Out-of-Hospital Cardiac Arrest Outcomes (OHCAO) registry.
- Interview ambulance clinicians to understand the challenges they face and to identify any perceived ambiguity in the existing guidance.
- Interview emergency department (ED) clinicians who receive cardiac arrest patients from the ambulance service to understand what factors influence their decisions to continue or to stop resuscitation.
- Undertake interviews with both survivors and relatives of non-survivors of cardiac arrest to understand their lived experience.
- Draft an updated TOR guideline suitable for adoption by national organisations.

The updated TOR guideline was developed iteratively over several phases, outlined below.

### Phase 1: understanding the ambulance service perspective

In order to understand the challenges faced by ambulance services, we engaged with JRCALC, NASMeD, AACE and Resuscitation Council UK (RCUK). At the request of JRCALC, NASMeD and AACE, we collaborated with the Ambulance Service Lead Paramedics Group (ASLPG) to progress the work.

Due to the limitations imposed by COVID-19, the ASLPG suggested the most appropriate approach would be to utilise an online questionnaire, developed in collaboration with an ASLPG member. The questionnaire was circulated to all UK ambulance services (Supplementary 2). Reminder emails were sent at four, eight and twelve weeks.

### Phase 2: evaluating the existing evidence and policy documents

Websites of relevant professional organisations (JRCALC, AACE, RCUK, ERC, International Liaison Committee on Resuscitation (ILCOR), Health and Care Professions Council UK (HCPC), General Medical Council (GMC), Nursing and Midwifery Council (NMC), Royal College of Paramedics, Faculty of Prehospital Care Royal College of Surgeons Edinburgh (FPH RCS Ed), British Association of Immediate Care (BASICs), Royal College of Emergency Medicine (RCEM), British Medical Association (BMA), Academy of Medical Royal Colleges (AoMRC) and Royal College of Nursing (RCN)) were searched to identify documents pertaining to resuscitation and end-of-life decisions.

The core content of retrieved documents was tabulated to generate a comprehensive list of potential topics for consideration in the updated guideline. This core content was cross-referenced with the content of the existing verification of death.

### Phase 3: systematic review and meta-analysis

We completed a diagnostic test accuracy meta-analysis of TOR rules following Cochrane Screening and Diagnostic Tests Methods Group recommendations (Deeks et al., 2023). Results were presented according to the Preferred Reporting Items for a Systematic Review and Meta-analysis of Diagnostic Test Accuracy Studies (PRISMA-DTA) Statement (McInnes et al., 2018).

### Phase 4: modelling performance of TOR rules

We modelled performance of 29 TOR rules identified in the systematic review using data from the OHCAO Registry. We were unable to model all the identified TOR rules as several required data that was not collected by the OHCAO Registry, for example EtCO_2_ trend. We compared the outcome predicted by the TOR rules with the true outcome to determine if any TOR rule could be used by paramedics to inform their decision making.

### Phase 5: interviews

We consulted a patient and public involvement (PPI) group (Clinical Research Ambassadors Group (CRAG), University Hospitals Birmingham NHS Foundation Trust), to determine what questions it would be acceptable to ask of a relative of a cardiac arrest victim who had died, how we should approach them, the content of the invitation letter / participant information sheet, as well as the timing of our approach.

The ambulance service initiated contact with survivors /relatives of non-survivors between three and six months after the cardiac arrest event. A letter explained that researchers at Warwick University were conducting a study to better understand the experience of cardiac arrest survivors and relatives of non-survivors and invited their participation.

When a potential participant responded, a researcher provided additional details of the study and sought verbal consent. An interview was scheduled, and an information pack was sent to the participant.

On the day of the interview, formal consent to participate was obtained. Interviews addressed the experience of the cardiac arrest event ‒ what happened, where, when, who was there, what the ambulance crew did and what happened afterwards. We sought their reflections on the event – expectations, dilemmas, challenges, values and beliefs. Finally, we explored what they would want others to learn from the event.

Interviews with ambulance and ED clinicians additionally utilised clinical vignettes, designed in collaboration with ambulance service partners, to examine contentious issues with respect to TOR (Supplementary 1). We sought to better understand clinician attitudes, beliefs and preferences.

### Phase 6: developing the initial draft guideline

The first draft of the new guideline was developed iteratively, informed by evidence generated in previous work streams and feedback from the ASLPG.

Briefly these steps included:

- Identification of potential topics for inclusion in the guideline, informed by the document analysis described Phase 2.
- ASLPG members voted on which topics to include in draft 1.0.
- Draft 1.0 was produced by MAS, guided by the ASLPG vote. The primary sources for reference were the existing verification of death guideline and the document analysis completed in Phase 2.
- Draft 1.0 was circulated to ASLPG members for feedback. Reviewers were asked to rate each section. Rating comprised two components: (i) how important they perceived the section to be and (ii) how contentious the evidence was. This latter aspect was particularly important for prioritising topics for discussion at the consensus conference (Phase 5). In addition to the scores, there was a free-text option for reviewers to feed back any preferred text or areas requiring clarification or expansion.
- Draft 2.0 was produced by MAS, informed by the ASLPG feedback, and was the document distributed for the consensus phase of development.

### Phase 7: developing consensus

We held a consensus conference for stakeholders, employing a nominal group technique (NGT) to identify, prioritise and achieve consensus on issues around which there was uncertainty or a need to balance risks and benefits. Attendees included representatives of AACE, JRCALC, JRCALC Guidelines Committee, NASMeD, ALSPG, RCUK, FPHC RCS Ed, BASICs, RCoP, RCoP Student Council, RCN, St John Ambulance, Royal Life Saving Society (RLS), Royal National Lifeboat Institute (RNLI), Search and Rescue, Wilderness Medicine, RCEM and National Ambulance Service Research Group (NASRG), clinical leaders from each ambulance service, clinical educators, research paramedics, ambulance paramedics, ambulance technicians, representatives of end-of-life and palliative care groups and clinical academics, as well as patient and public representatives.

Two weeks prior to the conference, attendees were sent copies of Draft 2.0 and any relevant supporting documents, such as summaries of evidence pertaining to clinical frailty scores, that might inform their deliberations on the day of the consensus conference.

At the conference an overview of all work packages completed in the course of the study was presented. Thereafter the audience divided into predetermined work groups. In these small groups a researcher facilitated discussion of key topics. We utilised flipcharts to annotate these discussions. A scribe (medical student trained volunteer) took comprehensive notes. On conclusion of the small group discussion, attendees reconvened, and facilitators provided plenary feedback on discussions within their respective groups. This was followed by plenary discussion, addressing topics raised by the attendees, and this was recorded by scribes in the same manner as for small group work.

The consensus process concluded with an audience vote on the importance of each topic to be included in the guideline. Flipcharts and scribe notes from the small group and plenary discussion would inform the content of each topic in the next draft of the guideline.

### Phase 8: updating the draft guideline to reflect the consensus opinion

Draft 3.0 was produced by MAS with reference to the consensus conference voting and the notes taken by scribes. Draft 3.0 was then circulated within the PROTECTeD team and to a selection of clinicians for internal review. Minor edits were made in response to feedback. Draft 3.1 was then circulated to key stakeholder organisations (JRCALC Guidelines Committee, NASMeD, RCUK, FPHC RCS Ed) for their formal comment. Draft 4.0, the draft of the TOR guideline, was produced by MAS in response to feedback from key stakeholders. Draft 4.0 of the guideline was distributed to a selection of clinically operational paramedics to obtain feedback on readability and usability (Draft 5.0).

### Phase 9: adapting the updated guideline for inclusion in JRCALC/AACE guidelines

Draft 5.0 of the guideline formed the starting point for discussion by the TOR guideline writing group. Two face-to-face meetings were convened, with additional work being undertaken via email discussion and by a smaller core writing team. The draft guideline (5.0) was distilled into two separate guidelines – the first described the factors to consider when making TOR decisions, while the second described the actions to be taken when verifying death, following TOR.

## Results

### Phase 1: understanding the ambulance service perspective

Questionnaires were completed by all English ambulance services, the Scottish Ambulance Service and the Welsh Ambulance Service. All ambulance services empower ambulance technicians and registered healthcare professionals to recognise conditions unequivocally associated with death. Several ambulance services also allowed lower grades (e.g. emergency care assistant (ECA) or community first responder (CFR)) to recognise conditions unequivocally associated with death.

All ambulance services empowered registered health care professionals to discontinue resuscitation in asystole. A small number of services also empowered ambulance technicians to discontinue resuscitation in asystole.

All ambulance services except one reported that registered health care professionals were empowered to discontinue resuscitation in PEA. Most services required clinicians to seek senior clinical advice prior to discontinuing; however, one service empowered all registered healthcare professionals to autonomously make the decision to stop in PEA.

Several ambulance services reported that registered health care professionals, predominantly doctors and critical care paramedics, were empowered to discontinue resuscitation in shockable rhythms (pulseless ventricular tachycardia (pVT) or ventricular fibrillation (VF)) when there had been no response to advanced life support. Services that allowed other paramedics to terminate resuscitation in shockable rhythms normally required that they seek senior advice prior to discontinuing.

All ambulance services recognised the risk posed to staff from transporting with CPR in progress. There was a strong desire to minimise unnecessary transport to mitigate this risk and to ensure adequate resources were available to respond in the community. All ambulance services recognised that management of PEA was complex and advocated for guidelines that empowered ambulance clinicians to stop resuscitation in PEA where clinically appropriate.

### Phase 2: evaluating the existing evidence and policy documents

We identified several key documents that informed content of the new TOR guideline. Key topics were extracted from each document and tabulated.

### Phase 3: systematic review and meta-analysis

Our meta-analysis identified very low-certainty evidence from 43 observational studies spanning 11 countries (Smyth et al., 2024). No randomised controlled trials were identified. Within these 43 studies, 15 reported the derivation of 20 unique TOR rules, 33 studies reported external data validations of 17 TOR rules and only one study described the clinical validation of a TOR rule (Table 1).

**Table 1.** Study type.

Author (year)	Derivation study	External data validation study	Clinical validation study
Bonnin (1993)	•		
Cheong (2016)		•	
Chiang (2013)		•	
Chiang (2017)	•		
Cone (2005)		•	
Diskin (2014)		•	
Drennan (2014)		•	
Fukada (2014)		•	
Glober (2019)	•		
Glober (2021)		•	
Goto (2019)	•	•	
Grunau (2017b)		•	
Grunau (2019)		•	
Harris (2021)		•	
Haukoos (2004)	•		
House (2018)	•	•	
Hreinsson (2020)		•	
Hsu (2022)		•	
Jabre (2016)	•		
Jordan (2017)		•	
Kajino (2013)		•	
Kashiura (2016)		•	
Kim (2015)		•	
Lee (2019)	•	•	
Lin (2022)		•	
Marsden (1995)	•		
Matsui (2023)		•	
Morrison (2007)	•	•	
Morrison (2009)		•	
Morrison (2014)			•
Ong (2006)		•	
Ong (2007)		•	
Park (2023)		•	
Petrie (2001)	•		
Sasson (2008)		•	
Shibahashi (2020)	•		
Skrifvars (2010)		•	
Smits (2023)		•	
SOS-Kanto (2017)	•	•	
Verbeek (2002)	•		
Verhaert (2016)		•	
Yates (2018)		•	
Yoon (2019)	•	•	

Our analysis showed that the majority of studies were undertaken within systems with very low survival. Modelling performance of identified TOR rules at 8%, 10% and 12% survival indicated a substantial increase in missed survivors (Smyth et al., 2024).

### Phase 4: modelling performance of TOR rules

We modelled the performance of 29 TOR rules using data from the OHCAO registry. We were unable to model the performance of 14 TOR rules, as they required data not available in the OHCAO database, for example EtCO_2_. We were limited to modelling performance using patients transported with CPR in progress, as these were the only population where we could reliably ascertain the proportion of false positives (TOR rule recommends TOR, but the patient survives).

Our analysis suggests four of the TOR rules (GLOBBER1, GOTO1, KOCARC3 and MARSDEN) correctly identified survivors 99% of the time; however, these rules recommended TOR in fewer than 10% of cases, limiting their utility. Three of the TOR rules (ERC, HAUKOOS1 and HAUKOOS3) recommended terminating resuscitation for would-be survivors between 45% and 55% of the time. Our analysis suggests existing TOR rules would not assist paramedics making decisions to transport or not.

### Phase 5: interviews

We conducted interviews with 14 relatives of non-survivors. Although three survivors made initial contact with the university, none consented to being interviewed. We also conducted 31 interviews with ambulance clinicians and 24 interviews with ED clinicians.

Our analysis identified that cardiac arrest is a traumatic event for relatives, with many participants describing feelings symptomatic of post-traumatic stress disorder. During resuscitation, relatives needed information from paramedics about what was happening but were also able to provide information about their relative’s wishes. Participants needed to know that everything possible was done to save their relative and were reassured when they could witness some of the resuscitation. Relatives were surprised how long resuscitation lasted, and some were distressed that it lasted so long.

With respect to clinicians, our analysis highlighted that ambulance clinicians frequently felt confident that the outcome of a resuscitation was very likely to be poor (that the patient would die or have very poor quality of life), but regularly felt that they were obliged to continue due to the constraints of the verification of death guideline and uncertainty about whether they were ‘allowed’ to make such decisions (e.g. stopping resuscitation when quality of life is likely to be very poor). Some ambulance clinicians expressed that they felt an obligation to continue resuscitation when some favourable factor (e.g. young age) was present, regardless of the clinical circumstance (e.g. no bystander CPR and continuous asystole despite over 30 minutes advanced life support) and their belief that outcome was likely to be poor. Hospital clinicians did not express concerns with ambulance clinician decisions to transport to hospital.

### Phases 6–9: developing an updated national ambulance guideline

The draft guideline was developed iteratively across phases, being refined at each stage and benefitting from wide stakeholder input. The final draft version of the guideline (5.0) is included in Supplementary 2. Feedback from operational clinicians was positive, in particular with respect to the algorithm, the ability to better make patient-centred decisions (including the ability to stop in all underlying rhythms) and the advice to delay formally recognising that death has occurred until five minutes after resuscitation was stopped. The updated JRCALC guidelines are pending publication and national roll-out.

## Discussion

The new TOR and verification of death guidelines were developed through synthesis of existing guidelines, policies and position statements, meta-analysis of published research, modelling the performance of alternative TOR rules and co-development with stakeholders. The draft guideline (5.0) was refined over several iterations. Changes were made following consultation with clinical leaders, interviews, a consensus workshop, stakeholder organisations and finally usability testing.

Stakeholder engagement increases the likelihood of adoption and implementation of guidelines (Armstrong et al., 2018). With respect to clinical guidelines, potential stakeholders include all organisations with an interest in the guideline and those who may be affected by it. Stakeholders therefore include health professionals, patients and their representatives, education and training bodies, as well as organisations responsible for the delivery of care (Cluzeau et al., 2012). We sought to engage stakeholders from a diverse range of organisations, clinical backgrounds and roles, members of the public who may receive treatment according to this guideline, individuals with healthcare education and research backgrounds, as well as organisations responsible for national policy and governance.

### Limitations

The evolution of this guideline has been limited to the views and insights of those who participated in the various phases of development. The initial search for documents that might inform the content of the guideline was conducted by MAS. Although every effort to conduct a comprehensive search was made, potentially significant documents may have been missed, and some topics worthy of inclusion may have been omitted. Although PPI participation was actively sought during different phases of development, these voices were not the dominant group. Although we feel we remained true to the insight of PPI contributors, the guideline may be skewed towards the viewpoint of health care professionals and ambulance clinicians in particular, as these were the dominant contributing group. We adopted an NGT approach to achieve consensus on what should be included in the guideline. Although this approach is well recognised as an effective method to achieve group consensus (Harvey & Holmes, 2012), it may be limited by its rigid method, which can stifle the discussion of potentially important inter-related topics (Vahedian-Shahroodi et al., 2023).

## Conclusions

The TOR and verification of death guidelines support ambulance clinicians to make best-interest decisions on behalf of patients who have suffered a cardiac arrest but not responded to resuscitation. These guidelines were primarily developed for use within ambulance services; however, they will be valuable for other services providing care to patients outside hospital. Establishing consistent practice across out-of-hospital care services will help to reduce variation in care, support the development of standardised education programmes for healthcare professionals and encourage the adoption of consistent documentation across services.

## Acknowledgements

We are indebted to the research teams at our participating sites, who identified potential participants and invited them to collaborate in our study. We are grateful to the clinicians and relatives who agreed to be interviewed and share their insights. Your contribution to this work has been invaluable. Members of the ASLPG made an essential contribution to the development of the draft TOR guideline. Participants at the consensus conference helped to shape the TOR guideline towards its final form. Finally, we are grateful to AACE, NASMeD, JRCALC, ALSPG, FPHC RCS Ed and RCUK for supporting this work as it has developed.

## Author contributions

MAS, GDP, FG, A-MS, RL and SP worked on the concept and design of this study. MAS, RL, FM and SP were responsible for statistical analysis. Qualitative analysis was undertaken by A-MS, FG, CH, KE and GG. MAS drafted the manuscript and all other authors contributed to critical review of the manuscript for important intellectual content. MAS acts as the guarantor for this article.

## Availability of datasets

Requests for data will be considered by the University of Warwick Clinical Trials Unit Data Sharing Committee six months following the publication of the official NIHR synopsis report (see NIHR Journal Library). Data from the meta-analysis will be provided in the form of contingency table data only. Ambulance service data will be anonymised. Requests for data from the OHCAO registry will require a separate request to University of Warwick Clinical Trials Unit Data Sharing Committee. We did not obtain specific consent to share qualitative data; therefore the Data Sharing Committee will only consider release of this data in exceptional circumstances, and then only in line with published guidelines. All data will be anonymised.

## Conflict of interest

GP is supported by the National Institute for Health Research (NIHR) Applied Research Collaboration (ARC) West Midlands and was a member of CTUs funded by the NIHR, COVID-19 Reviewing, NIHR CTU Standing Advisory Committee, HTA Clinical Evaluation and Trials Committee.

## Ethics

A favourable ethical opinion was obtained from the East Midlands ‒ Derby Research Ethics Committee (19/EM/0358).

## Funding

The PROTECTeD study was funded by the NIHR Health Services and Delivery Research (HS&DR) programme (17/99/34). The funder played no role in the design of the study, data collection, statistical analysis, interpretation of findings or reporting of results.

## Statement of generative AI in scientific writing

The authors did not use a generative artificial intelligence (AI) tool or service to assist with preparation or editing of this work.
